# Supplementary material for: Identification of photoperiod-induced specific miRNAs in the adrenal glands of Sunite sheep (Ovis aries)
Source: Front Vet Sci. 2022 Jul 22;9:888207. doi: 10.3389/fvets.2022.888207 (PMC9354845; doi:10.3389/fvets.2022.888207)
Supplement: Supplementary file 1 [file Data_Sheet_1.ZIP › Supplementary file/miRDeep2 file.zip!/miRDeep2 file/Know_Structure/SPLP42A2/oar-mir-148a.pdf]

miRBase precursor	: oar-mir-148a
Total read count	: 2518603
oar-miR-148a read count	: 2514829
remaining reads	: 3774

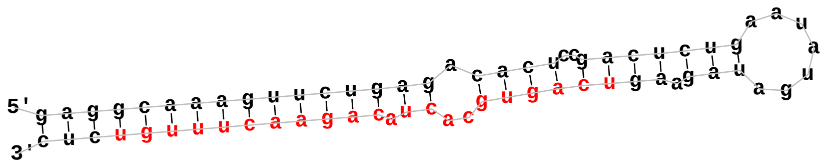

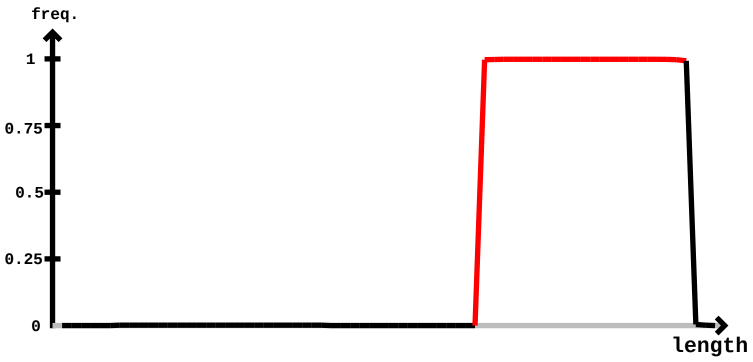

oar-miR-148a

```
5'- gaggcaaaguucugagacacuccgacucugaauaugauagaagucagugcacuacagaacuuugucuc  -3'    exp
    ((((((((((((((((.((((..(((((((.......))).))))))))..)).))))))))))))))   reads     mm         sample
    ....caaaguucugagacacuccga...........................................   7         0          seq
    ....caaaguucugagacacuccgac..........................................   3         0          seq
    ....caaaguucugagacacuccgacu.........................................   56        0          seq
    .....aaaguucugagacacucc.............................................   1         0          seq
    .....aaaguucugagacacuccg............................................   19        0          seq
    .....aaaguucugagacacuccga...........................................   93        0          seq
    .....aaaguucugagacacuccCa...........................................   1         1          seq
    .....aaaguucugagacacuccgac..........................................   58        0          seq
    .....aaaguucugagacacuccgacu.........................................   3519      0          seq
    .....aaaguucugagacacuccgacuc........................................   9         0          seq
    .....aaaguucugagacacuccgacucu.......................................   3         0          seq
    .....aaaguucugagacacuccgacucug......................................   1         0          seq
    ......aaguucugagacacuccgacu.........................................   3         0          seq
    ......aaguucugagacacuccgacucu.......................................   1         0          seq
    .........................................agucagugcacuacagaacu.......   1         0          seq
    .........................................agucagugcacuacagaacuuug....   1         0          seq
    ..........................................guUagugcacuacagaacuuugu...   1         1          seq
    ..........................................gucagugcacuacagaacuuugu...   81        0          seq
    ...........................................ucagugcacuacagaac........   15        0          seq
    ...........................................ucaguAcacuacagaacu.......   1         1          seq
    ...........................................ucaCugcacuacagaacu.......   5         1          seq
    ...........................................ucagugcacuacagaacu.......   299       0          seq
    ...........................................ucagugcacuacagaacuu......   621       0          seq
    ...........................................uUagugcacuacagaacuu......   2         1          seq
    ...........................................uUagugcacuacagaacuuu.....   7         1          seq
    ...........................................ucagugcacuacagaacuuu.....   3129      0          seq
    ...........................................uUagugcacuacagaacuuug....   12        1          seq
    ...........................................ucagugcacuacagaacuuug....   10366     0          seq
    ...........................................ucagugcacuacagaacuuugu...   2482584   0          seq
    ...........................................uUagugcacuacagaacuuugu...   3428      1          seq
    ...........................................uUagugcacuacagaacuuuguc..   8         1          seq
    ...........................................ucagugcacuacagaacuuuguc..   6914      0          seq
    ...........................................ucagugcacuacagaacuuugucu.   3531      0          seq
    ...........................................ucagugcacuacagaacuuugucuc   17        0          seq
    ............................................cagugcacuacagaacuuu.....   3         0          seq
```

oar-miR-148a

```
gaggcaaaguucugagacacuccgacucugaauaugauagaagucagugcacuacagaacuuugucuc
............................................cagugcacuacagaacuuug....    8       0       seq
............................................Uagugcacuacagaacuuugu...    12      1       seq
............................................cagugcacuacagaacuuugu...    1503    0       seq
............................................cagugcacuacagaacuuuguc..    10      0       seq
............................................Uagugcacuacagaacuuuguc..    1       1       seq
............................................cagugcacuacagaacuuugucu.    7       0       seq
.............................................agugcacuacagaacuu......    1       0       seq
.............................................agugcacuacagaacuuu.....    2       0       seq
.............................................agugcacuacagaacuuug....    13      0       seq
.............................................agugcacuacagaacuuugu...    2195    0       seq
.............................................agugcacuacagaacuuuguc..    9       0       seq
.............................................agugcacuacagaacuuugucu.    5       0       seq
.............................................agugcacuacagaacuuugucC.    1       1       seq
..............................................gugcacuacagaacuuug....    1       0       seq
..............................................gugcacuacagaacuuugu...    6       0       seq
...............................................ugcacuacagaacuuugu...    3       0       seq
................................................gcacuacagaacuuuUu...    1       1       seq
................................................gcacuGcagaacuuugu...    1       1       seq
................................................Acacuacagaacuuugu...    1       1       seq
................................................gcacuacagaacuuugu...    21      0       seq
................................................gcacuacagaacuCugu...    1       1       seq
................................................gcacuacagaacuuugG...    1       1       seq
```
